# Neurodoron® for Stress Impairments: A Prospective, Multicenter Non-Interventional Trial

**DOI:** 10.1155/2022/2626645

**Published:** 2022-02-25

**Authors:** Juliane Hellhammer, Katja Schmidt, Cristina Semaca, Rebecca Hufnagel

**Affiliations:** ^1^Contract Research Institute, Daacro, Trier 54296, Germany; ^2^Clinical Research, Weleda AG, Schwäbisch Gmünd 73525, Germany

## Abstract

**Introduction:**

Stress is associated with a multitude of physical and psychological health impairments. To tackle these health disorders, over-the-counter (OTC) products like Neurodoron® are popular since they are considered safe and tolerable. Experience reports and first studies indicate that Neurodoron® is efficient in the treatment of stress-associated health symptoms. To confirm this, a non-interventional study (NIS) with pharmacies was conducted.

**Methods:**

The NIS was planned to enroll female and male patients who suffered from nervous exhaustion with symptoms caused by acute and/or chronic stress. The main outcome measures were characteristic stress symptoms, stress burden, and perceived stress. Further outcome measures included perceived efficacy and tolerability of the product as assessed by the patients and collection of adverse drug reactions (ADRs). A study duration of about 21 days with a recommended daily dose of 3–4 tablets was set.

**Results:**

279 patients were enrolled at 74 German pharmacies. The analyzed set (AS) included 272 patients (mean age 44.8 ± 14.4 years, 73.9% female). 175 patients of the AS completed the NIS. During the study, all stress symptoms declined significantly (total score 18.1 vs. 12.1 (of max. 39 points), *p* < 0.0001). Furthermore, a reduction of stress burden (relative difference in stress burden, VAS = −29.1%, *p* < 0.0001) was observed. For most patients, perceived stress was reduced at the study end (PSQ total score decreased in 70.9% of the patients). 75.9% of the study population rated the product efficacy as “good” or “very good” and 96.6% rated its tolerability as “good” or “very good.” One uncritical ADR was reported. *Discussion/Conclusion*. This study adds information on the beneficial effects of Neurodoron® in self-medication. The results from this NIS showed a marked reduction in stress burden and perceived stress, along with an excellent safety profile of the medicinal product (MP) Neurodoron®. Further trials are required to confirm these results.

## 1. Introduction

The link between stress and a variety of physical and mental health disorders has been sufficiently proven by scientific evidence [1–6]. Thus, the WHO declares stress as one of the greatest health threats of the 21st century. Modern lifestyle, social media, and technologies such as digitalization, including the associated overstimulation, have made stress virtually omnipresent, regardless of culture and living environment [7, 8]. The numbers of stress-related costs regularly published by authorities and insurances speak for themselves and call for action [9–11]. In 2016, the German Opinion Research Institute forsa carried out a representative study on behalf of Techniker Krankenkasse, a statutory health insurance fund, according to which 60% of respondents in Germany perceive an increased stress burden within the past three years [12]. In the age group of 18 to 29-year-olds, this proportion was even as high as 75%. The same study confirms a particularly high correlation between stress and mental health.

In fact, stress is a physiologically sensible adaptation process to stimuli and impacts, helping the organism to respond as effectively and appropriately as possible. A psychological stress situation is usually characterized by novelty, uncontrollability, unpredictability, and the expectation of negative consequences that are personally relevant [13, 14]. Facing such a situation, the two central stress systems, the sympathetic nervous system, and the hypothalamic-pituitary-adrenal axis, are activated and orchestrate the organism accordingly while other systems, especially serotonergic and parasympathetic systems, are downregulated. While short-term stressors upregulate both stress systems followed by a normalization after the stressful situation comes to an end, chronic or extreme stress, as well as a certain stress vulnerability, can lead to a more permanent dysregulation of stress systems [1, 15] which in turn promotes diseases. While stress by itself is not a disease according to the criteria of the Diagnostic and Statistical Manual of Mental Disorders, Fifth Edition (DSM-5) [16], chronic stress favors a large number of diseases such as cardiovascular disease, diabetes, and skin and mental disorders. The first signs of stress-related health problems are typically nervousness, irritability, sleep disorders, headaches, and digestive problems, as well as anxiety disorders and depression [17–19]. Mental disorders, especially depression, anxiety, and adjustment disorders, are widespread [20] and often associated with particularly long downtime at work (in 2016 an average of 43 days [21]). A survey of students in the German federal state North Rhine-Westphalia has shown that stress often leads to the use of medication: one in two respondents said to be often or always stressed, complained of nervousness, fatigue, headaches, and sleep disorders. Here, every 10th student stated to fight stress with psychotropic drugs. A comparison of the prescribed daily doses showed a 55% increase between 2006 and 2010 [22]. Simultaneously, however, the proportion of people relying on complementary and alternative medicine (CAM) or dietary supplements increased when facing stress [23]. There are many different effective CAM options for the treatment of stress and other psychiatric disorders, ranging from music therapy to tai chi to phytotherapy [24–27], to mention some of the main ones. Herbal, homeopathic, and anthroposophic medicines are also very popular, as they are generally considered safe and are widely used in various indications [28–33].

Anthroposophic medicine commonly uses mineral substances for therapeutic use. Beyond naturally occurring minerals (e.g., quartz), the category also includes purified substances such as metals possibly having undergone specific treatment (e.g., gold prepared as “metallic mirror” by distillation under vacuum), inorganic compounds (e.g., potassium diphosphate), and “compositions” (e.g., Ferrum-Quarz), which are the result of complex preparation processes involving several mostly mineral-inorganic starting materials, not unlike preparations used in ayurvedic and Siddha medicine or in homeopathy (e.g., Causticum). These “mineral” substances are described in anthroposophic medicine and pharmacy textbooks and the Anthroposophic Pharmaceutical Codex. Once prepared as starting material, they are usually potentized according to processes described in anthroposophic and official homeopathic pharmacopoeias [34, 35].

In anthroposophic therapy, mineral remedies are of particular significance because they are the farthest removed from the living processes of the human organism. The MP of this NIS, Neurodoron®, is one of them. The integration of the mineral remedies in the organism requires the involvement of stronger organic forces than are needed for the integration of vegetal or animal substances, whose composition and structure are closer to that of the human organism. These forces originate in the I-organization, the hierarchically uppermost organizational level of the human organism, of spiritual nature, that works in all metabolic processes and enables the human being to harbor individual reflective consciousness. As a result, mineral remedies are considered to have a deeper and more durable action than vegetal or animal-based remedies [36–39].

For Neurodoron®, two clinical studies were performed between 2008 and 2012, indicating that the anthroposophic drug can be an efficient treatment for stress-related health problems [40, 41]. A first multicenter NIS conducted by practicing physicians in Germany from 2008 to 2009 [40] investigated the effect of the MP in participants reporting stress-related nervous fatigue. After an average of 46 days of Neurodoron® therapy, a significant reduction of all 39 recorded symptoms was observed. In the period between 2011 and 2012, a double-blind, placebo-controlled RCT with Neurodoron® [41] followed to demonstrate the efficacy in participants with nervous fatigue following diagnostic criteria defined in ICD-10 [42]. Significant improvements were observed for the symptoms of nervousness and irritability. Furthermore, the majority of the symptoms tested showed favourable trends in the Neurodoron® group when compared with the placebo group.

The following NIS should complement the data based on research with Neurodoron® gathered from both the previous RCT and NIS. Since the drug is predominantly used in self-medication, this prospective, pharmacy-based NIS should add information about the real-life use of the product.

## 2. Methods

### 2.1. Design

This prospective, pharmacy-based, multicenter NIS was conducted to collect data on the use of Neurodoron® regarding efficacy and safety in the approved indication. The NIS was notified to the German Federal Institute for Drugs and Medical Devices in September 2014 and was coordinated by the contract research organization (CRO) Winicker Norimed GmbH Medizinische Forschung/Germany. Two study visits were scheduled, a baseline visit and a final assessment on day 21:

(1) At baseline/day 1/beginning: when purchasing Neurodoron®, customers were asked whether they would like to take part in a NIS. If interested, the pharmaceutical staff checked the inclusion and exclusion criteria, explained the course of the study, and carried out the baseline survey after the patient had signed the consent form. The following data were collected:
  - Demographic, anthropometric, socioeconomic, and general data
  - Reason for the use of Neurodoron®
  - Previous therapy for neurasthenia
  - Intake of Neurodoron®
  - Stress symptoms
  - Stress burden (visual analog scale, VAS)
  - Perceived stress questionnaire (PSQ)
(2) Second assessment/day 21/end: towards the end of the 21 days study period, the patients received the end-of-study documentation by mail with the request to fill it out and return it directly to the CRO. The end-of-study documentation included the recording of the following parameters:
  - Intake of Neurodoron®
  - Subjective evaluation of Neurodoron®
  - Further therapeutic measures
  - Stress symptoms
  - Stress burden (VAS)
  - PSQ
  - Subjective assessment of efficacy and tolerability

Throughout the course, (suspected cases of) ADRs were recorded.

### 2.2. Patients

To be included in the study, patients had to have self-reported symptoms of nervous fatigue due to acute and/or chronic stress. No strict age limits were set. The preferred age range was 25 to 49 (due to the expected peak of cases in the study indication). Patients with known hypersensitivity to wheat starch, lactose intolerance, present pregnancy, or lactation were excluded from participation unless their doctor had explicitly recommended the use of Neurodoron®. All study patients were informed orally and in writing about the NIS and gave their written informed consent to participate. The NIS was notified to the German Federal Institute for Drugs and Medical Devices (BfArM) in accordance with § 67 para. 6, Sentence 1 AMG (German Arzneimittelgesetz (German Drug Law)).

### 2.3. Medicinal Product

The MP has been on the market for about 60 years. It has been authorized under the name Neurodoron® as an anthroposophic medicine (AM) with the indication of nervous exhaustion and metabolic dysfunction (Commission C monograph [43], registration number 6646311.00.00). One tablet contains the following active ingredients: 83.3 mg *Aurum metallicum praeparatum trituration* (*trit.)* D10, 83.3 mg *Kalium phosphoricicum trit*. D6, 8.3 mg *Ferrum-Quarz trit.* D2 (excipients: wheat starch and lactose). According to the market authorization, the daily intake of 3–4 tablets over the course of the day is recommended. The tablet can either be left to dissolve in the mouth or can be taken with liquid. Neurodoron® is taken for the duration of the NIS, which is 21 days.

### 2.4. Measurement of Study Parameters

#### 2.4.1. Demographic, Anthropometric, Socioeconomic, and General Data

Age, gender, height, weight, smoking status, marital status, and professional situation, including sector, were recorded.

#### 2.4.2. Reason for the Use of Neurodoron®

Patients were asked if they were suffering from acute or chronic stress or a combination of both. They could mark several reasons such as professional situation, exam, family situation, multiple burden/time deficit, and others as the origin of their perceived stress.

#### 2.4.3. Previous Therapy for Neurasthenia and Further Therapeutic Measures during the Study

Drugs that had already been used as a treatment for stress-related symptoms should be indicated and assessed regarding their efficacy and tolerability. Furthermore, questions related to a previous Neurodoron® therapy were asked, e.g., efficacy and safety were assessed on a rating scale (“1 = very good” to “4 = unsatisfactory” or “5 = I do not know”). At the end of the study, patients were asked whether they took other drugs besides the MP or whether they pursued other non-therapeutic measures as, e.g., psychotherapy, stress improvements techniques, sports, and/or yoga/pilates.

#### 2.4.4. Intake of Neurodoron®

If patients were using Neurodoron® for the first time, the start of use during the study was documented (i.e., at the earliest on the date of patient consent). If Neurodoron® was already taken before the start of the study, the respective start of intake was noted. In addition, the patients were asked about the dosage.

#### 2.4.5. Stress Symptoms and Symptom Sum Score

The patients rated the following 13 characteristic stress symptoms on a four-level Likert scale from “0 = absent” over “1 = mild,” “2 = moderate” to “3 = severe”: irritability, restlessness, nervousness, listlessness, depressive mood, mood swings, anxiety states, troubles to concentrate/lack of concentration, headache, sleep disorders, digestive disorders, muscular pain/tensions, fatigue. From the symptom scores, an additional sum score was calculated, which could reach a maximum value of 39 points.

#### 2.4.6. Stress Burden (VAS)

The patients assessed their stress burden on a 100 mm VAS [44]. The VAS is a widely accepted procedure with a bipolar scale ranging from “0 = not stressed at all” to “100 = maximally stressed.”

#### 2.4.7. Perceived Stress (PSQ)

In the present study, the revised German version of the PSQ consisting of 20 items was used [45]. The PSQ is a validated tool for assessing the individual perception, assessment, and processing of stressors. Respondents indicate on a scale from “1 = almost never” over “2 = sometimes,” “3 = often” to “4 = usually” how frequently they experience certain stress-related feelings such as feeling frustrated or tense. The PSQ provides results for the four subscales “worries,” “tension,” “joy,” and “demands,” in addition to a total score. For calculation of the total score, the values for “joy” are inverted. Thus, higher total scores indicate greater levels of stress. In order to assess scale values and a PSQ index representing the overall perceived stress, mean values are calculated from the raw item scores and linearly transformed to values between 0 and 100.

#### 2.4.8. Subjective Evaluation of Neurodoron®

As a final evaluation of the MP, patients were asked whether they would continue with the MP treatment and whether they would recommend the product (“yes,” “no,” “I do not know,” respectively). In addition, patients indicated their satisfaction with the treatment on a scale from “very satisfied” over “satisfied,” “unsatisfied” to “very unsatisfied” or “not specified.” Impact of the MP on the development of the disease was also rated (“great,” “small,” “not specified”).

#### 2.4.9. Subjective Assessment of Efficacy and Tolerability

Patients rated on a scale from “very good” over “good,” “satisfactory” to “unsatisfactory” or “I do not know” how they assessed the efficacy and tolerability of the MP.

#### 2.4.10. Safety/ADRs

Pharmacies informed patients at baseline that suspected cases of ADRs shall be reported separately by the patient to the pharmacy. These ADRs were documented by the pharmacies and redirected immediately (i.e., within 24 hours) to the sponsor for further action. Rating options regarding relatedness of the ADR to the MP were “certain,” “likely,” “possible,” “unlikely,” “unknown,” “not assessable.”

### 2.5. Statistical Calculations

Details of this descriptive evaluation were specified within a statistical analysis plan before database lock. All outcome measures were analyzed based on the AS. The AS includes all patients who met all inclusion and no exclusion criteria and had at least one intake of Neurodoron®. Missing values were not replaced. For the exploratory statistical analyses, only complete records were used. Statistical outliers were not excluded. Stress symptoms were tested with Wilcoxon signed-rank tests, while stress burden and perceived stress according to PSQ were analyzed using a paired *t*-test. Two-sided tests with an alpha level of 0.05 were performed. Statistical analyses were performed using SAS Version 9.2. Means of variables were reported as mean values ± standard deviation (SD) or standard error (SE) and were calculated using the AS.

## 3. Results

This multicenter NIS was conducted at a total of 74 pharmacies in Germany. The baseline documentation of the first patient was done on September 29, 2014, and the final documentation of the last patient was done on May 9, 2016. Out of 279 recruited patients, 272 (97.5%) patients were assigned to the AS (6 patients violated at least 1 exclusion criterion, 1 patient had not signed the consent form). 175 patients (64.3%) of the AS completed the NIS.

### 3.1. Demographic, Anthropometric, and Socioeconomic Data

The mean age was 44.8 years. More female (73.9%) than male persons (25.4%) participated in this study. The demographic, anthropometric, and socioeconomic data of the 272 patients are summarized in Table 1. 61.4% of the patients were married or in a relationship, 24.3% were single, and 14.0% were separated, divorced, or widowed. The majority of patients indicated that they were working (71.7%). Most worked in the social sector (39.3%).

### 3.2. Reason for the Use of Neurodoron®

When asked about the reasons for the use of Neurodoron®, the patients mainly reported a combination of acute and chronic stress (82.7%). As the most common causes of acute stress, patients (*N* = 258) reported the professional (58.1%) or familial (35.3%) situation. Chronic stress (*N* = 239) was also mostly based on the professional situation (56.1%), followed by multiple burden/time deficit (38.5%), the family situation (37.7%), and persistent psychological/emotional distress (33.9%).

### 3.3. Previous Therapy for Neurasthenia and Further Therapeutic Measures during the Study

When asked about concomitant therapies, 23.5% of patients said they were taking other medications (most frequent: 6.3% cardiovascular drugs, 5.9% systemic hormone preparations) and 49.3% used at least one non-drug therapy (28.7% sport, 20.6% stress management techniques, 10.7% yoga or pilates).

144 patients (52.9%) had previous treatment with other drugs against stress-related disorders. Efficacy of the best previous treatment was rated as “good” or “very good” by 60.4% of the patients, while 78.5% of the patients rated the tolerability of the previous treatment as “good” or “very good.”

### 3.4. Intake of Neurodoron®

Most patients followed the recommended dosage of 3–4 tablets per day (*N* = 220 in the beginning and *N* = 135 in the end of the NIS). 33.5% (*N* = 91) of the patients already used Neurodoron® before participating in the NIS. The study duration and correspondingly the intake duration of Neurodoron® varied widely between patients. The mean individual study duration was 37 ± 31 days (median: 29 days, range: 9–290 days, *N* = 153), the mean Neurodoron® intake duration during the NIS was 36 ± 34 days (median: 28 days, range: 8–289, *N* = 121). The overall mean Neurodoron® intake, including the intake before the NIS, was 100 ± 214 days (median: 32 days, range: 14–1751, *N* = 109).

### 3.5. Efficacy/Safety

#### 3.5.1. Stress Symptoms and Symptom Sum Score

**Figure 1:**
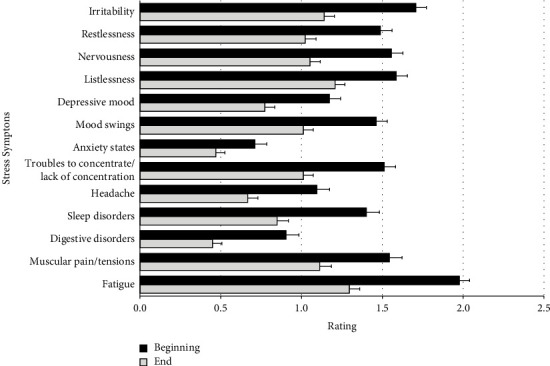
Changes in the 13 stress symptoms shown as mean and SE (N between 166 and 172, respectively) after a median study time of 29 days; “0 = absent,” “1 = mild,” “2 = moderate,” “3 = severe.”

All symptoms showed a significant improvement during the study (all *p* < 0.0001), with the most noticeable symptoms being fatigue (Δ = −0.7 ± 1.0), irritability (Δ = −0.6 ± 0.9), and sleep disorders (Δ = −0.5 ± 1.0). The results for all 13 stress symptoms are shown in Figure 1. The mean total score of stress symptoms for patients with values for both study visits was 18.1 ± 5.9 at baseline and 12.1 ± 6.4 at study end (max. total score: 39). Thus, there was a significant improvement of −6 ± 6.5 points (*p* < 0.0001, *N* = 170).

#### 3.5.2. Stress Burden (VAS)

**Figure 2:**
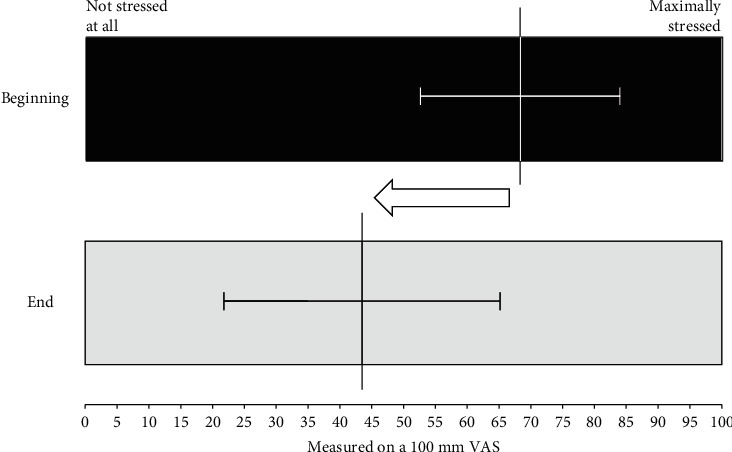
Changes in stress burden measured on a 100 mm VAS shown as mean ± SD (*N* = 166) after a median study time of 29 days; “0 = not stressed at all,” “100 = maximally stressed.”

For stress burden VAS data of 166 patients were available at both study visits. These patients showed mean values of 68.3 ± 15.7 at the beginning and 43.5 ± 21.6 at the end of the study (shown in Figure 2). This corresponds to a significant improvement of the stress burden by -29.1% ± 86.3% (*p* < 0.0001).

#### 3.5.3. Perceived Stress (PSQ)

**Figure 3:**
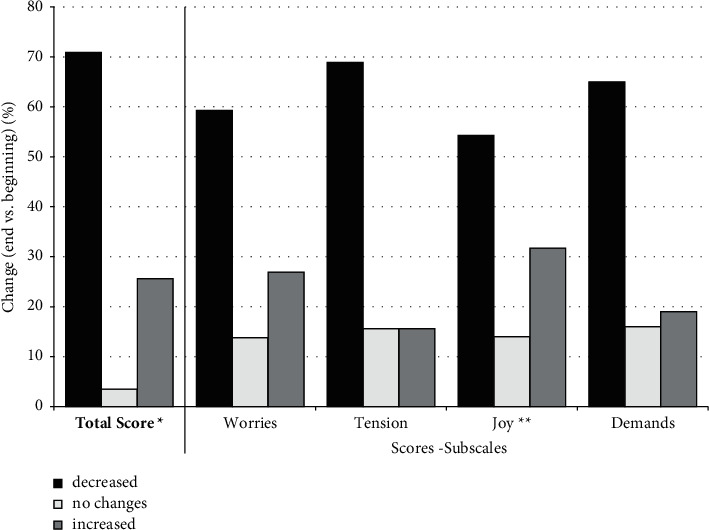
Changes in perceived stress according to PSQ in percent (N between 163 and 172, respectively) after a median study time of 29 days. ^*∗*^*“Joy” values were inverted for calculation of total score.*^*∗∗*^*“Joy” values are shown inverted for better understanding.*

In the majority of patients, perceived stress decreased during the study, as reflected in the total score. For the three subscales, “worries,” “tension,” and “demands,” the values decreased in most patients. For the subscale “joy,” the values predominantly increased compared to study start, i.e., the patients experienced more joy compared to baseline. The total score for perceived stress decreased in 70.9% of the patients. In 3.5%, there was no change and in 25.6%, the total score increased (shown in Figure 3).

#### 3.5.4. Subjective Evaluation of Neurodoron®

61.7% of patients who reached the end of the study (*N* = 175) reported they would continue with the therapy, and 82.9% of patients would recommend the Neurodoron® therapy. 86.9% of the patients were “satisfied” or “very satisfied” with the treatment, and 45.7% rated its influence on the disease as “great” (29.1% answered with “not specified” or data was missing).

#### 3.5.5. Subjective Assessment of Efficacy and Tolerability

**Figure 4:**
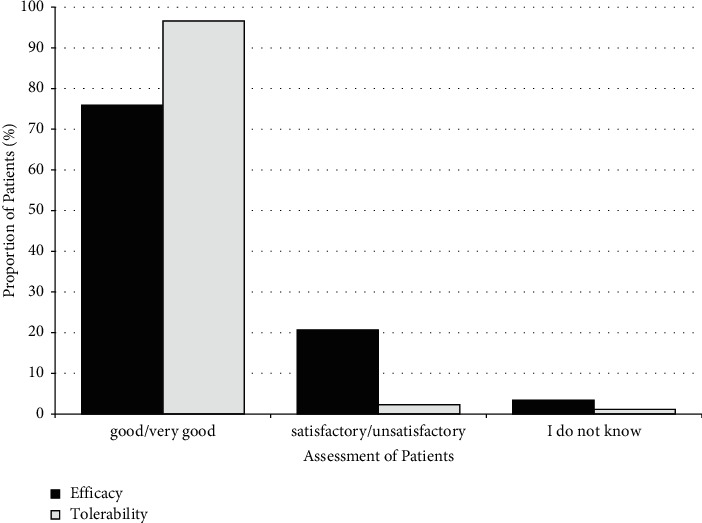
Subjective assessment of the efficacy and tolerability of Neurodoron® at study end (*N* = 174); assessment options: “very good,” “good,” “satisfactory,” “unsatisfactory,” and “I do not know.”

Based on the data of 174 patients, efficacy was rated as “good” or “very good” by 75.9% of patients at the end of the NIS, while 96.6% of patients rated the tolerability of Neurodoron® as “good” or “very good” (shown in Figure 4).

#### 3.5.6. Safety/ADRs

One ADR was reported, which was abdominal pain and nausea. As a potential reason, intolerance to lactose, an excipient of Neurodoron®, was considered. As other reasons (e.g., diet) could not be excluded, the relatedness with the MP was classified as “possible.”

## 4. Discussion

While stress and its associated symptoms are increasingly prevalent in today's society [7], the importance of an efficient treatment is increasing. Aside from, psychotropic drugs, herbal, homeopathic, and AM are very popular in fighting stress-related health impairment.

AM sees itself as integrative medicine, which extends the so-called university medicine to holistic aspects of the concept of disease and complementary medical approaches in the fields of drugs and various forms of art therapy and external applications [46]. In the physician-based multicenter NIS from 2008/2009 [40], 300 patients with stress-related nervous fatigue were analyzed, a good third of whom also had a burnout diagnosis. 272 patients with stress-related nervous fatigue were analyzed in the present pharmacy-based NIS. The proportion of women was comparable, as was the mean age (78.0% and 50.3 years (physician-based NIS) vs. 73.9% and 44.8 years (pharmacy-based NIS)). As expected from the design of the studies, the mean intake duration of Neurodoron® of 42.0 days was longer in the physician-based NIS than in this NIS with 36.0 days (treatment duration was set at 6 weeks for the physician-based NIS compared to 21 days in this NIS.). For the present NIS, the documentation at the study end did not take place at a fixed date in the pharmacy but was completed by the patient at home. Therefore, a deviation from the planned treatment duration of 21 days was to be expected. On average, the documentation was completed by the patients significantly later resulting in a longer treatment duration. One explanation for that fact could have been satisfaction with the preparation, both in terms of efficacy and safety; details are as follows.

Patients of the physician-based NIS showed a significant reduction of all 39 recorded symptoms such as irritability, headaches, and sleep disorder. The double-blind RCT that followed in 2011/2012 [41] to demonstrate the efficacy in patients with nervous fatigue following diagnostic criteria defined in ICD-10 [42] showed similar results. While there was no statistically significant difference to the placebo in the symptom sum score, 10 of the 12 symptoms tested in a post hoc analysis showed favourable trends in the Neurodoron® group compared to the control group. Statistically significant improvements were observed for the symptoms of nervousness and irritability. Consistent with the results of the first two studies conducted with Neurodoron® (physician-based NIS and RCT), this pharmacy-based NIS also showed that the MP can successfully reduce stress burden and stress-related symptoms such as fatigue, irritability, and sleep disorders in the OTC environment typical for Neurodoron®. Patients also reported a clear improvement in their perception of tension and worries and improved joy and better coping with the demands placed on them. When comparing the patients' subjective assessment of efficacy and safety, data from both the physician-based and the pharmacy-based NIS are very similar (efficacy/safety evaluation of Neurodoron® “good” or “very good”: physician-based NIS 78.7%/95.7% [40], pharmacy-based NIS 75.9%/96.6%).

Regarding efficacy, in the present NIS, the positive patient assessment of the product was also reflected in the fact that 86.9% were “satisfied” or “very satisfied” with the treatment, 75.9% rated the efficacy as “good” or “very good,” and 82.9% would recommend the treatment. The results of the two NISs provide consistent efficacy findings.

Regarding safety, the noteworthy good safety assessment by patients was confirmed by only one reported ADR within the entire study duration of one and a half years (with abdominal pain and nausea an uncritical ADR that was considered possibly related to the product). This shows, in summary, an exceptionally good safety profile of the product.

Real-life use observed in non-interventional studies brings out well the strengths of anthroposophic medicinal products. NISs are therefore a good way to complement a randomized controlled trial with its strict prerequisites. For example, patients in the RCT were not allowed to take any other medication for stress-related symptoms and were asked not to change their habits (daily activities, non-drug therapies, etc.). In contrast, the anthroposophic approach recommends a holistic therapy that also introduces lifestyle changes to improve the participant's quality of life. As the results of nervous exhaustion are manifold, individual therapy with several simultaneous measures is common practice. The individual therapeutic approach considers the patient within his/her entire life situation. The positive results of this and the previously conducted NIS reflect common treatment practice insofar more adequately. By this holistic approach, for instance, sleep and fatigue improve, which then indirectly facilitate regeneration of the activation systems, which is reflected in decreasing nervousness and irritability.

The goal of physicians and pharmacists is to offer individual treatment. A suitable target group would be patients who experience themselves as nervous, irritable, and inwardly restless and wish for more serenity and sovereignty in everyday life. It is conceivable that these individuals, in particular, will benefit from Neurodoron®, whereby the preparation could also be used as an adjunct to meditation and relaxation procedures.

There are limitations that should be considered for the interpretation of the data. The NIS was uncontrolled; therefore, confirmation of efficacy based on hypothesis-testing was out of scope. As Neurodoron® is a drug that emerges from AM, the mostly individualized and multimodal therapy concepts in the fields of AM make it more difficult to capture efficacy because of absent randomization and blinding possibilities. Controlled clinical trials provide important information that is complemented by non-interventional study results. The value of non-interventional studies lies in particular in their good external validity, as they allow insights into the reality of medical practice or, in this case, self-medication [47, 48].

Additionally, a certain selection bias for enrollment in the NIS was present due to the design of the study. Only patients who sought pharmacy advice, were open to CAM and were willing to participate in a clinical study were enrolled. These conditions may have introduced gender and social status bias with an unknown effect on NIS results. One might also speculate that patients are seeing a physician at a more advanced stage of the disease, which might also impact the results. However, there were good reasons for the chosen design. Neurodoron® is an OTC product and is mainly used for self-medication. Therefore, a pharmacy-based NIS may provide even better insight into the main patient population than a physician-based NIS. Overall, a certain selection bias cannot be excluded.

In general, stress impairments fluctuate in their intensity as they strongly depend on the increase or decrease of the stressors and the ability of the affected person to cope with the stressful situation. In the present NIS, patients with acute and/or chronic stress qualified for study participation. As the majority of patients (82.7%) reported to have a combination of both acute and chronic stress, it can be assumed that a rather stable and permanently ongoing stress level was present. Therefore, we consider it very unlikely that the improvement in stress symptoms can be exclusively explained by a sudden decrease or even disappearance of stressors. Together with the results of previous studies with Neurodoron®, these results suggest a positive effect of the treatment. However, as this NIS was uncontrolled, it is not possible to quantify the influence of treatment with Neurodoron® vs. fluctuation of individual stressors or adaption of coping strategies with respect to the observed outcome.

Lastly, the effect of Neurodoron® treatment might have been even underestimated in the NIS because patients also qualified for the NIS if they already had taken Neurodoron® before the study started (33.5% of patients). It could be assumed that these patients had benefited from Neurodoron® treatment and therefore wanted to continue it. As there are no baseline data without Neurodoron® treatment available for these patients, it cannot be ruled out that the improvement that was observed during the NIS was underestimated. This circumstance was deliberately accepted in order to get the whole picture of self-medication with Neurodoron® in pharmacies.

## 5. Conclusion

The results of this study complement the picture that has emerged from the previous studies: Neurodoron® seems to stabilize the balance between activation and recovery systems and to strengthen stress resilience. Neurodoron® appears to be a beneficial option for the treatment of nervous exhaustion and other stress-related symptoms either as monotherapy or as a component of a holistic treatment approach. What remains to be done is to support the present results with data from interventional and non-interventional trials, also in selected patient populations.

## Figures and Tables

**Table 1:** Demographic, anthropometric, socioeconomic, and general data of the patients.

Variable		All patients (*N* = 272)
Age, years	Mean ± SD	44.8 ± 14.4
	Range	16.0–88.0
	Missing data	3

Gender, N (%)	Male	69 (25.4)
	Female	201 (73.9)
	Missing data	2 (0.7)

Height (cm)	Mean ± SD	170.1 ± 8.9
	Range	150.0–202.0
	Missing data	4

Weight (kg)	Mean ± SD	71.9 ± 15.7
	Range	42.0–125.0
	Missing data	4

Smoking status, N (%)	Nonsmokers	212 (77.9)
	Smokers	55 (20.2)
	Missing data	5 (1.8)

Marital status, N (%)	Single	66 (24.3)
	Married	136 (50.0)
	In a relationship	31 (11.4)
	Separated	13 (4.8)
	Divorced	13 (4.8)
	Widowed	12 (4.4)
	Missing data	1 (0.4)

Professional situation	Working	195 (71.7)
	Unemployed	8 (2.9)
	Pupils/students/trainees	20 (7.4)
	Retired	35 (12.9)
	Housewife/househusband	11 (4.0)
	Missing data	3 (1.1)

Sector	Social sector	107 (39.3)
	Service	30 (11.0)
	Trade	17 (6.3)
	Industry/digital economy	15 (5.5)
	Craft sector/trade	10 (3.7)
	Administration/commercial area	25 (9.2)
	Other	13 (4.8)
	Missing data	55 (20.2)

## Data Availability

The data that support the findings of this study are not publicly available due to containing information that could compromise the privacy of research participants. Disclosure of data would not be in accordance with the European General Data Protection Regulation.

## References

[1] McEwen B. S. (2017). Neurobiological and systemic effects of chronic stress. *Chronic Stress*.

[2] Hellhammer D. H., Hellhammer J. (2008). Stress. The brain-body connection. *Key Issues in Mental Health*.

[3] Cohen S., Janicki-Deverts D., Miller G. E. (2007). Psychological stress and disease. *JAMA*.

[4] Dantzer R. (2018). Neuroimmune interactions: from the brain to the immune system and vice versa. *Physiological Reviews*.

[5] de Kloet E. R., Joëls M., Holsboer F. (2005). Stress and the brain: from adaptation to disease. *Nature Reviews Neuroscience*.

[6] O’Connor D. B., Thayer J. F., Vedhara K. (2021). Stress and health: a review of psychobiological processes. *Annual Review of Psychology*.

[7] Bhagat R. S., Segovis J., Nelson T. (2012). *Work Stress and Coping in the Era of Globalization*.

[8] Fischer T., Reuter M., Riedl R. (2021). The digital stressors scale: development and validation of a new survey instrument to measure digital stress perceptions in the workplace context. *Frontiers in Psychology*.

[9] Andlin-Sobocki P., Jonsson B., Wittchen H.-U., Olesen J. (2005). Cost of disorders of the brain in Europe. *European Journal of Neurology*.

[10] Heuer L. (2016). *Burnout und psychische Belastungen am Arbeitsplatz. Ursachen, Folgen und Maßnahmen zur Prävention*.

[11] The Lancet Global Health (2020). Mental health matters. *Lancet Global Health*.

[12] Wohlers K., Hombrecher M. (2016). *Entspann Dich, Deutschland: TK-Stressstudie*.

[13] Mason J. W. (1968). A review of psychoendocrine research on the sympathetic-adrenal medullary system. *Psychosomatic Medicine*.

[14] Hellhammer D. H., Stone A. A., Hellhammer J., Broderick J., Koob G. F., Le Moal M., Thompson R. F. (2010). Measuring stress. *Encyclopedia of Behavioral Neuroscience*.

[15] Russell A. L., Tasker J. G., Lucion A. B. (2018). Factors promoting vulnerability to dysregulated stress reactivity and stress-related disease. *Journal of Neuroendocrinology*.

[16] American Psychiatric Association (2018). *Diagnostic and Statistical Manual of Mental Disorders*.

[17] Busch M. A., Maske U. E., Ryl L., Schlack R., Hapke U. (2013). Prävalenz von depressiver Symptomatik und diagnostizierter Depression bei Erwachsenen in Deutschland. *Bundesgesundheitsblatt-Gesundheitsforschung-Gesundheitsschutz*.

[18] Nakao M. (2010). Work-related stress and psychosomatic medicine. *BioPsychoSocial Medicine*.

[19] Slavich G. M., Irwin M. R. (2014). From stress to inflammation and major depressive disorder: a social signal transduction theory of depression. *Psychological Bulletin*.

[20] Alonso J., Angermeyer M. C., Bernert S. (2004). Prevalence of mental disorders in europe: results from the European study of the epidemiology of mental disorders (ESEMeD) project. *Acta Psychiatrica Scandinavica*.

[21] Gesundheitsreport (2016). *Gesundheit zwischen Beruf und Familie*.

[22] (2013). Psychopharmaka gegen Stress. *Dtsch Aerztebl*.

[23] Eisenberg D. M., Davis R. B., Ettner S. L. (1998). Trends in alternative medicine use in the United States, 1990–1997. *JAMA*.

[24] Gorji N., Moeini R., Mozaffarpur S. (2021). On the therapeutic applications of music therapy in Persian medicine. *Traditional and Integrative Medicine*.

[25] Afrasiabian F., Mirabzadeh Ardakani M., Rahmani K. (2019). Aloysia citriodora Palau (lemon verbena) for insomnia patients: a randomized, double-blind, placebo-controlled clinical trial of efficacy and safety. *Phytotherapy Research*.

[26] Taylor-Piliae R. E., Morrison H. W., Hsu C.-H., Whitman S., Grandner M. (2021). The feasibility of tai chi exercise as a beneficial mind-body intervention in a group of community-dwelling stroke survivors with symptoms of depression. *Evidence-based Complementary and Alternative Medicine*.

[27] Gopukumar K., Thanawala S., Somepalli V., Rao T. S. S., Thamatam V. B., Chauhan S. (2021). Efficacy and safety of ashwagandha root extract on cognitive functions in healthy, stressed adults: a randomized, double-blind, placebo-controlled study. *Evidence-based Complementary and Alternative Medicine*.

[28] Akrami R., Hashempur M. H., Tavakoli A., Nimrouzi M., Sayadi M., Roodaki M. (2016). Effects of fumaria parviflora L on uremic pruritus in hemodialysis patients: a randomized, double-blind, placebo-controlled trial. *Jundishapur Journal of Natural Pharmaceutical Products*.

[29] Ghadimi M., Foroughi F., Hashemipour S. (2021). Randomized double-blind clinical trial examining the Ellagic acid effects on glycemic status, insulin resistance, antioxidant, and inflammatory factors in patients with type 2 diabetes. *Phytotherapy Research*.

[30] Hamre H. J., Glockmann A., Heckenbach K., Matthes H. (2017). Use and safety of anthroposophic medicinal products: an analysis of 44,662 patients from the EvaMed pharmacovigilance network. *Drugs-Real World Outcomes*.

[31] Schunk M., Le L., Syunyaeva Z. (2021). Effectiveness of a specialised breathlessness service for patients with advanced disease in Germany: a pragmatic fast-track randomised controlled trial (BreathEase). *European Respiratory Journal*.

[32] Stub T., Kristoffersen A. E., Overvåg G., Jong M. C., Musial F., Liu J. (2022). Adverse effects in homeopathy: a systematic review and meta-analysis of observational studies. *Explore*.

[33] Wagner P. J., Jester D., LeClair B., Taylor A. T., Woodward L., Lambert J. (1999). Taking the edge off: why patients choose St. John’s Wort. *Journal of Family Practice*.

[34] International Association of Anthroposophic Pharmacists IAAP (2020). *Anthroposophic Pharmaceutical Codex APC*.

[35] Meyer U., Pedersen P. A. (2017). *Anthroposophische Pharmazie*.

[36] Simon L. (2017). Relationship of the natural kingdoms to the four human entities. *Medizinische Sektion der Freien Hochschule für Geisteswissenschaft, Dornach/Switzerland–Gesellschaft Anthroposophischer Ärzte in Deutschland*.

[37] Schramm H. (2009). Chap. 3.2 Natursubstanzen und ihre Wirkungen. *Schramm H. Heilmittel der anthroposophischen Medizin: Grundlage–Arzneimittelporträts–Anwendung*.

[38] Husemann F., Wolff O. (1987). *Anthroposophical Approach to Medicine, Chap Mineral Remedies*.

[39] Glöckler M., Soldner G., Girke M., Glöckler G., Soldner (2020). Anthroposophische Arzneimittel mit Blick auf die funktionelle Dreigliederung und die Wesensglieder des Menschen. *Anthroposophische Medizin–Arzneitherapie für 350 Krankheitsbilder*.

[40] Rother C., Oexle J. (2010). Einsatz von Neurodoron bei Patienten mit nervöser Erschöpfung aufgrund von Stress. *Der Merkurstab*.

[41] Hufnagel R., Bergtholdt B., Hellhammer J., Semaca C., Schnelle M. (2016). Effects of Neurodoron in patients with nervous exhaustion-results from a randomized controlled clinical trial. *European Journal of Integrative Medicine*.

[42] World Health Organization (1993). *The ICD-10 Classification of Mental and Behavioural Disorders–Diagnostic Criteria for Research*.

[43] Kommission C. (1999). Anthroposophische Arzneimittel. Aufbereitungsmonographien. BfArM. *Gesellschaft Anthroposophischer Ärzte (GAÄD) in Deutschland e.V. Filderstadt*.

[44] Bond A., Lader M. (1974). The use of analogue scales in rating subjective feelings. *British Journal of Medical Psychology*.

[45] Fliege H., Rose M., Arck P., Levenstein S., Klapp B. F. (2001). Validierung des “Perceived Stress Questionnaire” (PSQ) an einer deutschen Stichprobe. *Diagnostica*.

[46] Glöckler M. (2014). *Anthroposophische Arzneitherapie für Ärzte und Apotheker*.

[47] Anglemyer A., Horvath H. T., Bero L. (2014). Healthcare outcomes assessed with observational study designs compared with those assessed in randomized trials. *Cochrane Database of Systematic Reviews*.

[48] Mishra D., Vora J. (2010). Non interventional drug studies in oncology: why we need them?. *Perspectives in Clinical Research*.

